# Botulinum Toxin A and Osteosarcopenia in Experimental Animals: A Scoping Review

**DOI:** 10.3390/toxins13030213

**Published:** 2021-03-14

**Authors:** Min Jia Tang, H. Kerr Graham, Kelsey E. Davidson

**Affiliations:** 1 Department of Orthopaedics, The Royal Children’s Hospital, Parkville, VIC 3052, Australia; minjiatang29@gmail.com; 2 Department of Paediatrics, University of Melbourne, Parkville, VIC 3010, Australia; kerr.graham@rch.org.au; 3 Department of Orthopedics, Shriners Hospitals for Children, Chicago, IL 60707, USA

**Keywords:** Botulinum Toxin A, muscle atrophy, sarcopenia, osteopenia, experimental animals, cerebral palsy

## Abstract

We conducted a scoping review to investigate the effects of intramuscular injection of Botulinum Toxin A (BoNT-A) on bone morphology. We investigated if the muscle atrophy associated with Injection of BoNT-A had effects on the neighboring bone. We used the search terms: osteopenia, bone atrophy, Botulinum Toxin A, Micro-CT, mice or rat. The following databases were searched: Medline, Embase, PubMed and the Cochrane Library, between 1990 and 2020. After removal of duplicates, 228 abstracts were identified of which 49 studies satisfied our inclusion and exclusion criteria. The majority of studies (41/49) reported a quantitative reduction in at least one measure of bone architecture based on Micro-CT. The reduction in the ratio of bone volume to tissue volume varied from 11% to 81% (mean 43%) according to the experimental set up and study time points. While longer term studies showed muscle recovery, no study showed complete recovery of all bone properties at the termination of the study. In experimental animals, intramuscular injection of BoNT-A resulted in acute muscle atrophy and acute degradation of the neighboring bone segment. These findings may have implications for clinical protocols in the use of Botulinum Toxin in children with cerebral palsy, with restraint recommended in injection protocols and consideration for monitoring bone density. Clinical studies in children with cerebral palsy receiving injections of Botulinum are indicated.

## 1. Introduction

Cerebral palsy (CP) is the most common cause of physical disability affecting children in developed countries with a prevalence of approximately 2 per 1000 live births [1]. Cerebral palsy is characterized by developmental delay, limitations in gross motor function, and alterations in muscle tone, most commonly hypertonia, including spasticity, dystonia, and mixed moment disorders [1]. The most commonly used medical intervention for hypertonia is intramuscular injection of Botulinum Toxin A (BoNT-A) to hypertonic muscles [1,2]. It is commonly stated that BoNT-A reduces spasticity in the injected muscle, but this is not a primary effect. Injection of BoNT-A produces chemo-denervation of skeletal muscle by blocking acetylcholine release at the neuromuscular junction for between six weeks and four months, which is followed by intramuscular nerve sprouting and return of muscle function [1,2,3,4]. Chemo-denervation causes acute muscle atrophy, and it is the muscle atrophy which results in a reduction in spasticity as a secondary effect of BoNT-A injection. Spasticity reduction is therefore a secondary effect, not a primary effect [1,2,5].

Studies in both typically developing human volunteers and small mammal models suggests that recovery from BoNT-A induced muscle atrophy is slow and may be incomplete at 12 months after injection [2,5]. Recent reviews have confirmed that muscle and bone development are impaired in children with CP, even in ambulatory children with milder forms of the disorder [1,6]. Given the close relationship between muscle morphology and physiology, and the morphology and physiology of the neighboring bone segment, some investigators have studied the effects of BoNT-A injection on the neighboring bone segment in animal models, including mice, Sprague Dawley rats and New Zealand white rabbits [2,6]. Although much of this research has only been published in the last 10 years, we previously reported consistent themes emerging which may have relevance to clinicians who inject BoNT-A for hypertonia in children with cerebral palsy [2,5]. In a review which focused on injection induced sarcopenia, we noted that some studies included references to osteopenia [5]. The relevant literature is extensive and heterogeneous in terms of experimental animals, injection protocols, target muscles and study duration [5,6]. Scoping reviews are particularly helpful when the literature is complex and heterogeneous, to understand the extent of knowledge and new concepts in an emerging field. We restricted our review to investigations in the hindlimb of experimental muscles of mice and rats as being potentially more relevant to the clinical situation, the management of hypertonic gait disorders in children with cerebral palsy [2,5]. We conducted a scoping review of the recent literature to report common themes on the effect of BoNT-A muscle denervation on bone morphology and draw these to the attention of clinicians who use BoNT-A and who may not be aware of the basic science research.

## 2. Results

**Figure 1:**
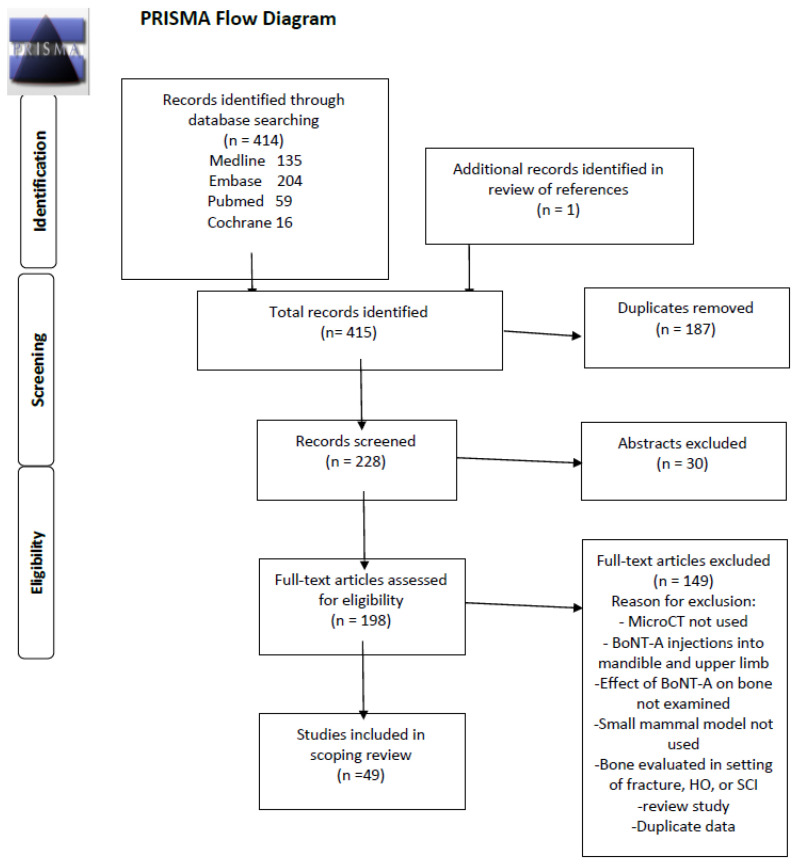
PRISMA Scoping Review Study Diagram.

From 228 abstracts identified meeting the inclusion criteria, 179 were removed based on the exclusion criteria, which resulted in 49 studies being included in the final review. (Figure 1) The studies included in this scoping review are summarized in Appendix A [7,8,9,10,11,12,13,14,15,16,17,18,19,20,21,22,23,24,25,26,27,28,29,30,31,32,33,34,35,36,37,38,39,40,41,42,43,44,45,46,47,48,49,50,51,52,53,54]. The type of small mammal used in the studies was fairly evenly split, with rats utilized in 20 studies and mice in 29. The animals ranged in age from 6-44 weeks, with the median age being 16 weeks, which is generally considered to be of adult age. The most commonly injected muscles were the quadriceps [8] and calf muscles [15,16], injected in 41 and 40 studies, respectively, and often both were injected [20,21]. A few studies also injected the hamstring and Tibialis anterior muscles [51]. Most studies evaluated the neighboring femur or tibia to the injected muscle, but two studies injected the quadriceps muscle and examined the bone properties of the tibia [28,53]. A weight-based dose of 2 U/100 g of BoNT-A was the most frequently used amount in 21 (43%) of studies, but there was heterogeneity in weight-based doses ranging from 0.6–2 U/100g. Some studies used a constant dose of BoNT-A for each animal, ranging from 0.5–4U. The most commonly used dose 2 U/100 g is equivalent to 20 U/kg, which is the upper limit of doses advised during BoNT-A therapy in children with CP receiving multilevel injections [1,2,5]. The most frequently used controls were an equivalent volume saline injection in a separate cohort of animals or the contralateral limb, with or without a saline injection. A few studies also compared the effects of BoNT-A paralysis to paralysis induced by suspension [29,30] or to Achilles tenotomy [15]. Most studies evaluated the effects of BoNT-A on bone at a single time point, though some studies examined the longitudinal effect of BoNT-A at multiple time points. The follow-up period ranged from 5–196 days, with a median time of 28 days.

All studies found significant changes in the neighboring bone segment, with reductions in bone density, architecture, and strength, to varying degrees [11,23,52]. (Appendix A) The average decrease in bone mineral density was 12% (SD 7%), while bone mineral content showed an average decrease of 18% (SD 9%). The greatest consequences to bone architecture were observed in trabecular bone, which resulted in significant decreases in BV/TV in both the metaphyseal (mean −42%) and epiphyseal (−46%) regions. (Table 1) Reduction in trabecular thickness appeared to have the most significant effect on bone tissue loss (mean −20%), though detrimental changes were often also seen in the trabecular number (−15%), trabecular spacing (+17%), and structural model index (+126%). Connectivity density showed wide variation across studies and overall showed no consequential change (+2%). When comparing the decrease in BV/TV in the studies that used juvenile small mammals (< 12 weeks of age) to those with adult animals, there was a greater loss seen in the juvenile animals (mean 55%) versus adult (38%). Cortical bone changes were evaluated in 32 articles, which were predominantly measured at the diaphysis with a few measurements taken from the metaphysis. (Table 2) There was a consistent decrease in cortical bone area (mean −11%) which was primarily the result of increased endosteal absorption with resultant cortical thinning (−12%) and expansion of the marrow area (+7%). In studies that examined changes in bone at a cellular and molecular level, it was noted that osteopenia was primarily mediated by a rapid increase in osteoclasts, and that genes related to bone formation were downregulated whilst genes related to bone resorption were upregulated [13,18,22,25,43]. Alterations in mechanical properties of bone were reported in 15 studies. There was a consistent loss of bone strength across all studies at both the femoral neck or metaphyseal region and the diaphysis [25,34,35,36,37,38,39,40,41,47,48]. However, the methodology and test conditions were too heterogeneous to permit quantitative group analysis.

All injected muscles demonstrated a significant decrease in cross sectional area and loss of muscle mass. The majority of studies found a maximum loss around 40–60% in muscle mass (range 18–81%) and cross-sectional area (range 16–56%). The muscles demonstrated rapid atrophy after injection, with the maximum effect noticed around three to four weeks. The timing of changes in muscle compared to bone is of interest in that muscle atrophy was noted to be present within a few days of injection and showed near complete recovery by three to four months. The effects on bone predominantly appeared after muscle and were slower to recover. Only one study evaluated the effects of a second BoNT-A injection performed after one month [35], and the longest follow-up was seven months, with median follow-up of one month. Given these limitations it is not possible to ascertain the long-term effects of a single injection episode or the effects of repeated BoNT-A injections.

A few studies examined the effects of BoNT-A on bone in differing situations. One example is a comparison of BoNT-A versus tail suspension, both of which result in a period of unloading on the experimental limb, but the use of BoNT-A not only greatly diminishes ground reaction forces [17] but also muscle contraction forces on the neighboring bone [29,30]. While Ellman et al. found that BoNT-A resulted in twice the loss of trabecular bone volume compared to tail suspension, Warden et al. demonstrated a greater decrease in trabecular bone volume with tail suspension [29,30]. However, both found that BoNT-A compounded the effects of trabecular and cortical bone loss when injected in addition to tail suspension. Manske et al. utilized Achilles tenotomy as another technique to induce non-weight bearing and compared this to BoNT-A injection with a sham surgery or BoNT-A plus tenotomy. They demonstrated a decrease in trabecular bone volume in BoNT-A compared with Achilles tenotomy, but no significant differences in bone properties between BoNT-A and BoNT-A in addition to Achilles tenotomy [15]. Furthermore, their findings demonstrate that the majority of bone loss in BoNT-A induced osteopenia can be attributed to its paralytic effect on neighboring muscle and that any direct effect of BoNT-A on bone, if present, would be negligible [15].

## 3. Discussion

In the quest for safe and effective spasticity management in children with cerebral palsy, Botulinum Toxin A appeared to offer many advantages [1,2]. BoNT-A tends to remain in the target muscle with relatively small amounts of systemic spread giving a largely local effect [2]. Multiple studies have shown a consistent reduction in Modified Ashworth Scale (MAS) and Modified Tardieu Scale (MTS) [2]. However, the change in muscle stiffness as measured by MAS and MTS may not be a specific effect on spasticity but the effects of chemo-denervation and muscle atrophy [5]. The combination of these effects on muscle consequently leads to decreased contractile forces on bone. Therefore, BoNT-A may not be an agent which results in focal reduction in spasticity but rather it produces focal denervation, focal muscle atrophy and focal changes in the neighboring bone [4]. Reduction in spasticity is a secondary phenomenon [2,5].

Previous reviews have found that the muscle atrophy which follows injection of BoNT-A in experimental animals is extensive, slow to recover, and may be accompanied by other adverse effects including fatty infiltration of muscle and upregulation of pathways leading to muscle fibrosis [2,5]. In both animal models and typically developing human volunteers, muscle deficits persist at one-year post injection [5]. This may have implications for injection frequency protocols in children with cerebral palsy given that in the only two RCTs to date examining injection frequency, both found injecting once per year was just as effective as injecting three times per year, for spastic equinus [55,56].

As well as unwanted effects in skeletal muscle, the animal studies reviewed in this scoping review have reported a consistent adverse effect of BoNT-A on bone morphology in small mammals. The pattern of bone loss following single time point injections of BoNT-A is rapid and acute, followed by a prolonged and often incomplete recovery [12]. This loss is primarily the result of muscle atrophy and decreased muscle contractile forces on the neighboring bone induced by BoNT-A chemodenervation. It is a cause for concern that BoNT-A injection in skeletal muscle in small mammals is so reliable in inducing acute, prolific bone loss, that it has become the model of choice for studying disuse osteopenia in animal experimentation [7]. In many of the studies in this review, injection of BoNT-A was used to test interventions for prevention of disuse osteopenia including vibration therapy and anti-resorptive drugs [9,10,13,14,19,20,21,24,26,31,33,34,37,38,39,41,42,47,48,51]. The animal studies are consistent in their findings, although the severity and localization of effects on bone vary with the injection protocol and age at injection.

Impairments of bone health are a major clinical problem in children with cerebral palsy at baseline, particularly those who are not ambulant [6]. However, bone health is also impaired in ambulant children, with obvious decreases in segmental bone length, bone width, and bone density in the affected limb of children with hemiplegia compared to their “less affected/unaffected” limb [5,6]. Given the widespread use of BoNT-A in children with cerebral palsy, with most guidelines recommending an injection frequency of approximately once every three to six months, it is concerning that bone properties have not fully recovered by six months after injection. This review does not allow us to report on the long-term effects of repeat BoNT-A injections on growing bone in small mammals, as the longest follow-up in this scoping review was 28 weeks and only one study evaluated the effect two BoNT-A injections given one month apart. However, there is a possibility that repeated injections might result in cumulative decrements in bone morphology, physiology and function. In addition, there is a gap in the animal literature investigating the long-term effects on bone maturation in skeletally immature bone, the period when BoNT-A is commonly utilized in children with CP. [1,2] It may be time to consider the effects not only on the injected muscle but on the accompanying bone segment [1,2]. Further investigation into the possibility of additional decrements to bone health as a result of BoNT-A use in children with cerebral palsy is warranted, as this scoping review clearly highlights the development of osteosarcopenia from a single injection in experimental small mammals. The strengths of our study are that we focused our attention on two small mammal species, injection of BoNT-A of hindlimb muscles only and all studies included bone property measurements with micro-CT. The weaknesses of our study primarily lie in the heterogeneity of the study protocols. The differences include variable injection protocols (dose, dilution and volume), differing controls, variable age of animal subjects, variations in study duration, as well as the timing and frequency of micro-CT measurements, and inclusion of other factors that affect bone measurements. However, we feel that this review has merit as it is the first scoping review to evaluate the effects of osteopenia caused by muscle chemical denervation from BoNT-A. While the osteosarcopenic effects of BoNT-A injection in small mammals cannot be directly correlated to humans, the findings highlight a need for monitoring muscle and bone morphology in children with cerebral palsy undergoing BoNT-A therapy, especially those receiving repeated injections.

## 4. Conclusions

We suggest from our review of the animal literature the need for caution in the use of BoNT-A in children with cerebral palsy, in light of long lasting, potentially non-reversible adverse effects on muscle and bone. Further encouragement to reduce the frequency of BoNT-A injections in children with cerebral palsy are the two RCTs that demonstrated no difference in outcomes between one or three injections per year [55,56]. It is not possible from the animal literature to date to extrapolate the harmful effects in children with cerebral palsy. However, it is reasonable to suggest that the monitoring of bone growth and bone density be considered as an important part of good clinical practice.

## 5. Materials and Methods

A literature search was performed in January 2020 using the following key terms of osteopenia, bone atrophy, Botulinum Toxin A, Micro-CT, mice or rat. (Table 3) The following databases were searched: Medline, Embase, PubMed and the Cochrane Library, between 1990 and 2020. The starting time point was chosen based on the period when BoNT-A first became commercially available, and investigation into its effect on bone and muscle increased. During early reviews of the BoNT-A literature, the authors became aware of studies in experimental animals examining osteopenia and developed a more refined search strategy based on an attempt to find parallels between the use of BoNT-A in experimental animal studies and its clinical use in ambulant children with cerebral palsy. This study was performed in accordance with the PRISMA-ScR (Preferred Reporting Items for Systematic reviews and Meta-Analyses extension for Scoping Reviews) guidelines. After removal of duplicates, 228 abstracts were identified for further review. Abstracts were reviewed by two authors (MT and KD) and any discrepancies in outcomes of screening were resolved in group discussion with all authors. Papers were selected for inclusion if they met the following criteria:

### 5.1. Inclusion Criteria

1. Experimental studies with a full manuscript published in English that addressed effects of BoNT-A injections on bone in the hindlimb of mice or rats.

### 5.2. Exclusion Criteria

1. Studies were excluded if BoNT-A injections were tested in non-small mammal animal models.
2. Studies were excluded if the site of muscle injection was not in the lower limbs (e.g., masseter muscle to study the effects on the mandible).
3. Studies were excluded if they did not include objective measurements of bone properties using micro-CT.
4. Studies were excluded if they evaluated the effect of BoNT-A on fracture healing or in spinal cord injury.

We chose to primarily analyze the effect of BoNT-A on bone properties with micro-CT to have an objective and uniform method of measurement across the studies [7]. There was great variability amongst the studies on which micro-CT properties were measured and provided objective results, so we elected to focus on the most commonly reported trabecular and cortical properties to improve statistical relevance. Micro-CT measurements were performed on a region of interest in either the tibia or femur of the small mammal. Trabecular variables were evaluated in the metaphysis or epiphysis, and cortical variables were most generally examined in the diaphysis with occasional measurements in the metaphysis. The measurements were compared to a control limb, which was commonly a saline injected or untreated limb in a control group, or the contralateral limb of the BoNT-A injected mammal. Less frequently, these measurements were compared to baseline (day 0) measurements. In studies that included other factors affecting bone properties, such as orchidectomy or lactation [8,9,10], we compared the BoNT-A injected limb to a similarly treated control. Many of the properties measured by micro-CT may be unfamiliar to some clinicians; the following is a description of the most commonly cited properties that were examined in this scoping review. 

### 5.3. Common Trabecular Measurements

1. Bone volume/tissue volume (BV/TV): percentage of total tissue volume (cancellous space) occupied by trabecular bone.
2. Trabecular thickness (Tb.Th): mean thickness of trabeculae.
3. Trabecular number (Tb.N): average number of trabeculae per unit length.
4. Trabecular separation (Tb.Sp): mean distance between trabeculae.
5. Structural model index (SMI): indicates the structure of trabecular bone in the form of cylindrical rods or parallel plates—where rods confer a weaker formation (higher SMI) than plates (lower SMI).
6. Connectivity density (CD): measure of the degree of connectivity of trabeculae (higher is stronger).

### 5.4. Common Cortical Measurements

1. Cortical thickness (Ct.Th): mean cortical thickness.
2. Cortical bone area (Ct.Ar): area occupied by cortical bone.
3. Total cross-sectional tissue area (Tt.Ar): total area inside the periosteal envelope.
4. Cortical area fraction (Ct.Ar/Tt.Ar): ratio of cortical bone to total tissue area inside periosteal envelope.
5. Cortical marrow area (Ma.Ar): area occupied by medullary tissue.

In addition to micro-CT variables, we evaluated bone measurements of bone mineral density (BMD) and bone mineral content (BMC) from DEXA scans and mechanical stress testing, as these provide a more comprehensive understanding of the effects on bone and are often utilized in orthopaedic literature. Muscle measurements, including mass and cross-sectional area (CSA), were also examined.

In studies that provided objective measurements of trabecular and/or cortical micro-CT properties, the percentage of change between the BoNT-A injected limb and the control limb was calculated to the nearest percentage for each of the variables provided. Data were analysed using Microsoft Excel for Mac, version 16.35 (Redmond, WA, USA). Mean and standard deviation were reported for the most commonly examined micro-CT variables, and for BMD and BMC DEXA measurements of the BoNT-A injected hindlimb. In studies that performed longitudinal measurements, the time point where the bone property showed the greatest difference was used for calculations in order to remove the effect of reconstitution over time. There was variability in the methods for muscle measurement, so ranges are provided for these measures. Descriptive statistics are given for mechanical testing.

## Figures and Tables

**Table 1:** Micro-CT trabecular bone property measurements.

	BV/TV	Tb.Th	Tb.N	Tb.Sp	SMI	CD
*N* (articles with objective measurements)	31	28	30	26	17	14
Average % (SD)	−43 (19)	−20 (11)	−15 (15)	17 (28)	126 (203)	2 (50)
Min %	−81	−46	−68	0	−10	−68
Max %	−11	4	9	164	800	163
*N* (articles with metaphyseal measurements)	30	27	29	25	16	14
Metaphyseal Average % (SD)	−42 (19)	−17 (14)	−16 (16)	20 (31)	42 (78)	−21 (23)
*N* (articles with epiphyseal measurements)	4	4	6	6	5	4
Epiphyseal Average % (SD)	−46 (15)	−28 (13)	-10 (8)	8 (5)	440 (222)	73 (48)

Bone volume to Total tissue volume (BV/TV), Trabecular thickness (Tb.Th), Trabecular number (Tb.N), Trabecular spacing (Tb.Sp), Structural model index (SMI), Connectivity density (CD), Standard deviation (SD).

**Table 2:** Micro-CT cortical bone property measurements.

	Ct.Th	Ct.Ar	Ma.Ar	Tt.Ar
*N* (articles with objective measurements)	17	18	11	14
Average % (± SD)	−12 (6)	−11 (7)	7 (5)	−2 (3)
Min %	−27	−25	−3	−6
Max %	−2	2	14	5

Cortical thickness (Ct.Th), Cortical area (Ct.Ar), Marrow area (Ma.Ar), Total tissue area (Tt.Ar), Standard deviation (SD).

**Table 3:** Full list of formal search terms for Medline.

Ovid Search Number	Search Terms
1	exp Botulinum Toxins/
2	(botox or botulinum or BTX-A or BTXA or BONT-A or BONTA or dysport or xeomin).tw,kf.
3	(rimobotulinum adj toxin adj b) or myobloc or (abobotulinum adj toxin adj a) or (onabotulinum adj toxin adj a) or (incobotulinum adj toxin adj a) or (letibotulinum adj toxin adj a) or (abobotulinumtoxin adj a) or (onabotulinumtoxin adj a) or (incobotulinumtoxin adj a) or (letibotulinumtoxin adj a)).tw,kf.
4	mouse or mice or rat or rats or murine).af.
5	osteoporosis/or bone resorption/or osteolysis/
6	“bones of lower extremity”/or exp leg bones/
7	Bone Density/
8	Bone Diseases, Metabolic/
9	(bone or bones or osteopenia or osteoporosis or osteolysis or femur or tibia).tw,kf,hw.
10	(1 or 2 or 3) and 4 and (5 or 6 or 7 or 8 or 9)
11	Limit 10 to (English language and yr = “1990–Current”)

## Data Availability

The summary of the articles reviewed is provided in Appendix A. Further information is available from the corresponding author.

## Abbreviations

BoNT-A	Botulinum toxin A
BMD	Bone mineral density
BMC	Bone mineral content
DEXA	Dual energy x-ray absorptiometry
CSA	Cross sectional area
BV/TV	Percentage of Bone volume to Total tissue volume
MAS	Modified Ashworth Scale
MTS	Modified Tardieu Scale
RCT	Randomized control trial

## Appendix A

**Table A1:** Summary of articles included in scoping review.

First Author/year	Species	Number	Follow Up (weeks)	Target Muscle(s)	BoNT-A Dose	Trabecular BV/TV Max. Loss	Cortical Area Max. Loss
Blouin 2006 [8]	Rat	56	12	Quad.	2 U	52%	N/A *
Warner 2006 [11]	Mouse	20	3	Quad, calf	2 U/100 g	54%	N/A
Grimston 2007 [12]	Mouse	16	12	Quad, ham, calf	2 U/100 g	Decreased **	16%
Grimston 2011 [13]	Mouse	40	19	Quad, calf	2 U/100 g	Decreased	Decreased
Liboubain 2008 [7]	Rat	48	4	Quad	2 U	30%	9%
Liboubain 2018 [14]	Mouse	21	12	Quad	2 U/100 g	64%	N/A
Manske 2010 [15]	Mouse	73	4	Calf	1 U/100 g	22%	8%
Manske 2010 [16]	Mouse	36	16	Calf	1 U/100 g	25%	4%
Manske 2011 [17]	Mouse	13	6	Calf	1 U/100 g	28%	N/A
Manske 2012 [17]	Mouse	27	3	Calf	1 U/100 g	Decreased	Decreased
Poliachik 2010 [18]	Mouse	20	2	Calf	2 U/100 g	81%	N/A
Poliachik 2014 [19]	Mouse	64	1	Calf	2 U/100 g	Decreased	N/A
Agholme 2011 [20]	Rat	40	4	Quad, calf	5 U	66%	N/A
Agholme 2011 [21]	Rat	48	4	Quad, calf	5 U	Decreased	N/A
Aliprantis 2012 [22]	Mouse	36	2	Quad, calf	2 U/100 g	51%	N/A
Bouvard 2012 [23]	Rat	25	5	Quad	1.5 U	55%	N/A
Sheng 2012 [24]	Rat	21	9	Quad	2 U	18%	8%
Thomsen 2012 [25]	Rat	108	4	Quad, ham, calf	1.7 U/100 g	11%	N/A
Bruel 2013 [26]	Rat	72	4	Quad, ham, calf	4 U	34%	N/A
Marcias 2013 [27]	Rat	80	1	Quad, calf	5 U	58%	N/A
Marchand-Libouban 2013 [28]	Mouse	40	4	Quad	2 U/100 g	46%	25%
Warden 2013 [29]	Mouse	40	6	Quad, ham, calf, Tib Ant	0.5 U	Decreased	9%
Ellman 2014 [30]	Mouse	40	3	Quad, calf	2U/100 g	66%	24%
Grubbe 2014 [31]	Rat	60	4	Quad, ham, calf	4 U	26%	N/A
Morse 2014 [32]	Mouse	20	2	Quad, calf	0.5 U	25%	N/A
Sandberg 2014 [33]	Rat	20	4	Quad, calf	5 U	74%	N/A
Vegger 2014 [34]	Rat	72	4	Quad, ham, calf	1.7 U/100 g	25%	N/A
Vegger 2015 [35]	Rat	57	8	Quad, ham, calf	1.7 U/100 g	24%	N/A
Vegger 2016 [36]	Mouse	35	3	Quad, calf	2 U/100 g	62%	15%
Vegger 2017 [37]	Mouse	48	3	Quad, calf	2 U/100 g	56%	18%
Vegger 2018 [38]	Mouse	42	3	Quad, calf	2 U/100 g	Decreased	20%
Vegger 2018 [39]	Mouse	48	3	Quad, calf	2 U/100 g	Decreased	16%
Lodberg 2015 [40]	Mouse	80	3	Quad, calf	2 U/100 g	60%	N/A
Lodberg 2018 [41]	Mouse	58	3	Quad, calf	2 U/100 g	Decreased	Decreased
Mabilleau 2015 [5]	Rat	14	4	Quad	2 U	17%	5%
Niziolek 2015 [42]	Mouse	24	3	Quad, ham, calf, Tib Ant	20 µL	35%	6%
Rucci 2015 [43]	Mouse	-	3	Quad, calf	2 U/100 g	Decreased	N/A
Bach-Gansmo 2016 [44]	Rat	77	28	Quad, ham, calf	1.7 U/100 g	N/A	N/A
Laurent 2016 [45]	Mouse	-	19	Quad, ham, calf	2 U/100 g	Decreased	8%
Canalis 2017 [46]	Rat	12	3	Quad, calf	2 U/100 g	59%	16%
Brent 2018 [47]	Rat	72	6	Quad, ham, calf	4 U	28%	8%
Omstrup 2018 [48]	Rat	72	6	Quad, ham, calf	4 U	Decreased	8%
Zhang 2018 [49]	Rat	42	8	Quad	2 U	46%	N/A
Bain 2019 [50]	Mouse	16	4	Calf	2 U/100 g	72%	N/A
Bullock 2019 [51]	Mouse	80	4	Quad, ham, calf, Tib Ant	20 µL	43%	N/A
Gatti 2019 [52]	Rat	24	4	Quad, ham, calf	4 U	18%	14%
Xu 2019 [53]	Rat	24	8	Quad	0.6 U/100 g	22%	N/A
Liphardt 2020 [54]	Mouse	33	2	Calf	2 U/100 g	Decreased	N/A
Sorensen 2020 [10]	Mouse	81	3	Rectus femoris, calf	2 U/100 g	57%	11%

* N/A: article did not report micro-CT measurements of this bone property. ** Articles that reported a statistical change in BV/TV in the manuscript text or in graphic format, but did not provide objective measurements, are indicated here subjectively. Botulinum toxin (BoNT-A), Bone volume to Total tissue volume (BV/TV), Hamstring muscle group (Ham), Tibialis anterior muscle (Tib Ant), Unit (U), gram (g), Liter (L).
